# Unconvincing support for role of mirror neurons in “action understanding”: commentary on Michael et al. (2014)

**DOI:** 10.3389/fnhum.2014.00553

**Published:** 2014-07-25

**Authors:** Caroline Catmur

**Affiliations:** Psychology, University of SurreyGuildford, UK

**Keywords:** mirror neuron, transcranial magnetic stimulation, action understanding, perception, premotor cortex, action perception

Mirror neurons, firing when an action is performed but also when the same action is perceived, have been the source of both excitement and controversy since their discovery (Di Pellegrino et al., 1992). One key to this polarization of opinion is the claim that mirror neurons are involved in “action understanding” (Di Pellegrino et al., 1992; Rizzolatti and Sinigaglia, 2010). As we have recently discussed (Cook et al., 2014), the term “action understanding” has not been clearly defined, and has been used to refer to various different psychological processes, including action perception (Saygin et al., 2004); identification of the goal of an action (Rizzolatti and Fabbri-Destro, 2008); and the computation of an actor's intentions in a particular context (Fogassi et al., 2005). This has contributed to confusion around what role, if any, mirror neurons may play in social interaction.

Data on the contribution of mirror neurons—or mirror neuron brain areas, in particular premotor cortex—to a psychological process that might possibly correspond to “action understanding” are relatively sparse. Some, but not all, patient data indicate that action *perception* may require mirror neuron areas (Saygin, 2007; Moro et al., 2008; but Arévalo et al., 2012, provide an alternative view; note also that the understanding of action *language* appears to require the basal ganglia, rather than somatotopically organized motor areas: Ibáñez et al., 2013). Transcranial magnetic stimulation (TMS) of premotor cortex disrupts aspects of action perception including visual discrimination of actions (Candidi et al., 2008), configural processing of bodies (Urgesi et al., 2007) and judgment of body esthetics (Calvo-Merino et al., 2010). It also impairs the ability to use information from perceived actions to judge the weight of grasped objects (Pobric and Hamilton, 2006) and to initiate on-line predictions about ongoing actions (Stadler et al., 2012). Thus, there is some evidence for a mirror neuron contribution to action *perception*, but little to support their involvement in a higher-level process of “action understanding” (see also Mikulan et al., 2014).

Therefore, Michael et al. (2014) addressed the twin questions of whether mirror neuron areas are involved in a process that might be termed “action understanding,” and whether this process corresponds to action perception or to something more akin to goal identification or computation of intentions. Using disruptive TMS to premotor cortex, they demonstrated that performance on an action processing task was affected in a body-part-specific way. Thus, disruption of a more dorsal part of premotor cortex, thought to correspond to an area involved in control of hand movements, impaired processing of observed hand, but not mouth actions; whereas disruption of a more ventral area, thought to control mouth movements, impaired processing of mouth, but not hand, actions. This result complements the existing literature by demonstrating not only that premotor cortex is involved in processing of others' actions, but that its involvement may be at the level of the motor program of particular body parts.

More interestingly, however, Michael et al. measured the processing of others' actions in three different ways. In all three tasks, participants saw a contextual cue, then watched a video of an action of the hand or mouth. Their task was to judge which of three subsequent probe items matched the action video they had observed. The “simple” task tested action perception: participants had only to perceive the action video and make a delayed match to sample judgment. The “intermediate” task tested both action perception *and* the ability to match an action to its goal object. Finally, the “complex” task tested action perception, the ability to match an action to its goal object, *and* the ability to select a context-appropriate goal object. It is apparent that the simple task requires only action perception, not action understanding, whereas the other tasks involve higher-level processes in addition to action perception. If premotor cortex is involved in higher-level processes of action understanding then the intermediate and/or complex tasks should be more impaired by TMS than the simple task.

TMS to premotor cortex impaired participants' performance, in a body-part specific way, on all three tasks, but crucially, there were no significant differences between tasks. Recall that all three tasks required action perception: since performance on all three tasks was impaired, these data suggest that mirror neuron areas are involved, *not* in higher-level processes such as matching an action to its goal object or selecting the relevant object for that action in a given context, but instead in a lower-level process of action perception. Michael et al. describe their results as demonstrating the role of mirror neuron areas in “action understanding”; instead, however, these data support the claim that premotor cortex may contribute to the *perception* of others' actions.

It does not appear, therefore, that these data are in contradiction with what Michael et al. term a “deflationary” account of mirror neuron properties: the suggestion that mirror neuron responses during action observation are the result of sensorimotor associations. If mirror neurons are the result of bidirectional associations between sensory and motor representations of actions (Heyes, 2001, 2010), this does not rule out the possibility that they may play a role in action perception (Cook et al., 2014). However, one relevant implication of the associative account for Michael et al.'s results is that TMS-induced disruption to motor representations of a particular body part might propagate, via such sensorimotor associations, to sensory representations of that body part, producing the observed impairments in action perception. A useful next step would be to combine TMS with neuroimaging, to reveal the brain networks affected by TMS in this study, and in particular whether premotor TMS influences sensory areas.

In conclusion, these data do not convincingly support the claim that mirror neurons are involved in high-level “action understanding”; if anything, they contradict this claim. However, they do demonstrate that premotor cortex may, in some circumstances, contribute to action perception. Future studies testing mirror neuron contributions to action *understanding* must therefore control for premotor contributions to action *perception*, as well as providing a clearer definition of what is meant by “action understanding.”

## Conflict of interest statement

The author declares that the research was conducted in the absence of any commercial or financial relationships that could be construed as a potential conflict of interest.
